# What is social about social perception research?

**DOI:** 10.3389/fnint.2012.00128

**Published:** 2013-01-25

**Authors:** Christoph Teufel, Elisabeth von dem Hagen, Kate C. Plaisted-Grant, James J. Edmonds, John O. Ayorinde, Paul C. Fletcher, Greg Davis

**Affiliations:** ^1^Brain Mapping Unit, Department of Psychiatry, Behavioural and Clinical Neuroscience Institute, University of CambridgeCambridge, UK; ^2^Cognition and Brain Sciences Unit, Medical Research CouncilCambridge, UK; ^3^Department of Psychology, Behavioural and Clinical Neuroscience Institute, University of CambridgeCambridge, UK

**Keywords:** social perception, social neuroscience, interaction, gaze perception, face perception, mental state attribution, theory of mind, autism

## Abstract

A growing consensus in social cognitive neuroscience holds that large portions of the primate visual brain are dedicated to the processing of social information, i.e., to those aspects of stimuli that are usually encountered in social interactions such as others' facial expressions, actions, and symbols. Yet, studies of social perception have mostly employed simple pictorial representations of conspecifics. These stimuli are social only in the restricted sense that they physically resemble objects with which the observer would typically interact. In an equally important sense, however, these stimuli might be regarded as “non-social”: the observer knows that they are viewing pictures and might therefore not attribute current mental states to the stimuli or might do so in a qualitatively different way than in a real social interaction. Recent studies have demonstrated the importance of such higher-order conceptualization of the stimulus for social perceptual processing. Here, we assess the similarity between the various types of stimuli used in the laboratory and object classes encountered in real social interactions. We distinguish two different levels at which experimental stimuli can match social stimuli as encountered in everyday social settings: (1) the extent to which a stimulus' physical properties resemble those typically encountered in social interactions and (2) the higher-level conceptualization of the stimulus as indicating another person's mental states. We illustrate the significance of this distinction for social perception research and report new empirical evidence further highlighting the importance of mental state attribution for perceptual processing. Finally, we discuss the potential of this approach to inform studies of clinical conditions such as autism.

## Introduction

Extensive networks within the primate visual system seem to be dedicated to the perceptual processing of social stimuli. For instance, classical electrophysiological work in macaques has identified distinct cell populations in inferior-temporal cortex that are specifically tuned to faces (Perrett et al., 1992; Tsao et al., 2006) and studies in humans using psychophysical and functional magnetic resonance imaging (fMRI) techniques have revealed dedicated mechanisms that process another person's facial expressions (Adolphs, 2002), their gaze-direction (Jenkins et al., 2006; Calder et al., 2007, 2008), or their facial identity (Webster and Macleod, 2011). Each of these studies provides core examples of what many researchers refer to as “social perception.” At first glance, this label seems obvious: the term social perception denotes those perceptual and sensory-motor processes that are tuned to and elicited by classes of objects encountered in social interactions. However, aside from this (trivially) obvious answer, the question of what the specific nature of stimuli is that elicits neural mechanisms of social information processing has so far not been adequately answered. In fact, studies in areas such as social perception, social cognition, or social cognitive neuroscience, that specifically focus on aspects of information processing that are supposedly dedicated to the social have faced the difficulty of defining their topic of research (e.g., Adolphs, 2010) and are vulnerable to criticisms that deny the existence of such dedicated mechanisms (e.g., Fernandez-Duque and Baird, 2005).

Here, rather than focusing our attention directly on previous empirical or philosophical contributions to this topic, we address a related, more specific and more tractable question, exploring the extent to which experimental stimuli typically used in the laboratory resemble social stimuli as they are encountered in the real world. We argue that it is important to distinguish at least two broad levels of resemblance: namely, the level of physical stimulus properties and the level of how the observer conceptualizes the stimulus in terms of mental states. We argue that this analysis has important implications for the types of questions different experimental designs are able to address. Second, we provide new data that illustrate the importance of distinguishing between the different levels in order to tease apart the various processes involved in social perception. Finally, we discuss the implications of this framework for social perception research with a specific focus on clinically relevant work.

As a point of departure, let us consider an observer's experience in a typical social perception experiment. They sit in a dark room performing a repetitive, pared-down task, pressing buttons in response to monochromatic, cropped images of socially-relevant stimulus features such as another's angry facial expressions, each presented on a monitor for a few hundred milliseconds. This type of experiment conventionally serves as a working model of human perception of others' angry faces in everyday viewing. However, with such a constrained set of stimuli, it can of course do so only to a limited degree. To illustrate, consider, by way of contrast with the imagined experiment, a heated exchange with a boss, colleague, or partner, in which you assessed the escalation of the situation in part by closely observing their increasingly angry facial expressions. Some aspects are shared between the two scenarios, and looking at a picture of an angry face presented on a screen likely recruits some the same perceptual mechanisms that viewing the angry face of a person *live* does. In both instances the observer has to perform a discrimination on the basis of subtle differences in canonical stimulus configurations. However, in a different sense the two scenarios bear little relation to one another.

First, there are clear *physical* differences between the stimuli: in everyday viewing, facial expressions dynamically change, they are embedded within the rich context of another's bodily actions and speech prosody, and cues from multiple information sources often indicate the same dimension of interest. These differences do need addressing—and partly have been started to be addressed—in social cognitive neuroscience, but they are not our key focus here. For the purposes of this analysis, we shall leave aside the clear physical differences between the stimuli. Rather, we address those differences that are not directly and locally stimulus-bound. In particular, the observer's knowledge that the other person is physically present likely engages perception in fundamentally different ways to the simple observation of a picture or movie in an experiment. It is these latter differences that make experimental “social” stimuli such as picture of a face in some important sense “non-social” as we will discuss in more detail below.

Social perception research has largely avoided tackling the difficult challenge of describing the different ways in which experimental stimuli can resemble real social stimuli. Yet, if its goal is to understand the perceptual information processes taking place when people interact with others rather than when they look at briefly presented pictures of other people, confronting this issue can no longer be postponed. Given the drastic differences in stimulus properties and in the deeper social dimension between most stimuli used in the laboratory and those existing in the real world, the assumption that the former directly correspond to any subset of the latter might be invalid. Simple images are undoubtedly a useful tool in face and body perception research, identifying those parts of the human visual system tuned to perceptual processing of socially-relevant stimulus features. However, experiments employing such stimuli address only certain aspects of what the visual system is faced with in everyday life and can at best provide a frame of reference from which to study real-world social interactions. It therefore seems crucial to develop a framework that allows for the evaluation of the types of perceptual information processing that can be studied with different types of stimuli. As a starting point for the development of such a framework, we believe that it is heuristically useful to distinguish between at least two different levels at which social stimuli can be conceptualized. These levels are linked by complex dependencies and interactions, and are directly related to different levels of information processing.

At the most fundamental level, other people are physical objects and information processing mechanisms should apply to their external spatiotemporal features in similar ways as to inanimate objects. Moreover, some social stimuli are distinguished from other object classes by specific canonical spatiotemporal configurations and may be processed in a manner accorded only to such social stimuli. Prime examples are faces or biological motion. These stimuli are social in the sense that we typically encounter their spatiotemporal properties only in social interactions. Translating this to the laboratory, at this level, experimental stimuli such as a picture of a face are conceptualized as “social” due their physical stimulus features, that resemble those with which an observer would typically interact. With certain caveats, pictures, animations, or videos are appropriate stimuli for studies interested in social perception at this level.

However, on a second level, stimuli as encountered in real social interactions differ fundamentally from those typically used in the laboratory by the fact that they are indicators of other peoples' mental states. Another person's smile is not just a change in the configuration of the spatiotemporal properties of the stimulus but indicates a deeper underlying social dimension, namely, the other's mental or emotional state. The use of such perceptual properties to attribute mental states to others is termed theory of mind (ToM; Adolphs, 2009; Apperly, 2011). Conventionally, studies address how social stimuli trigger ToM processes in a bottom–up manner (Frith and Frith, 2003; Nummenmaa and Calder, 2009) However, a pervasive problem for the interpretation of many of these studies arises from the fact that almost all of these experiments employ pictures, static animations or, at best, videos of faces and people as stimuli. Such stimuli are appropriate for testing social perception at the level of perceptual stimulus properties, but the degree to which they can trigger other socio-cognitive processes involved in real-life interactions is unclear. Whereas static pictures may often trigger some cognitive processes similar to ToM, there are obvious problems involved in equating looking at a picture or video with looking at a real person. When watching Tom Cruise on TV, the stimulus does not elicit embarrassment because a celebrity is present and you are sitting in your living room in your pajamas. As an observer you know and in some sense *perceive* that pictures and animations presented in an experiment, or even filmed actors, are, currently, objects that cannot see, feel or think. When using such stimuli in the laboratory, it is important to realize that they differ substantially from social stimuli encountered in everyday social interactions and represent, at best, ambiguous targets for mental-state attribution. This notion is supported by a small, but growing number of studies, some of which are reviewed below, that indicate that looking at a real person or at a stimulus the observer believes to be a real person can trigger qualitatively different information processing than observing pictures, animations, and videos of other people (Wohlschläger et al., 2003a,b; Teufel et al., 2009, 2010a,b; Pönkänen et al., 2011; Moore et al., in press).

A related difficulty in interpreting social perception studies arises from the fact that many experimental designs confound the two levels in a more subtle way. To illustrate, at a mental-state level, a person with open eyes can see and a person with closed eyes cannot. Human observers tend to interpret such stimuli purely in terms of the underlying mental states. Logically, however, equating open and closed eyes with the ability or inability to see is confounding the mental state itself with its possible, but not necessary, external manifestation. For instance, a blind person with open eyes cannot see, a person can smile without being happy, or cry without being sad; the observer's knowledge of the observed individual may serve to disambiguate these percepts. Accordingly, it is important to distinguish the mental state from the observable behavior that communicates it, not only logically, but also empirically in experiments. Conventional studies, using images or movies in limited semantic contexts, cannot do so. In other words, when using such stimuli to study social perception it is impossible to determine whether the perceptual differences or the different mental states indicated by the perceptual differences are responsible for differential processing.

We recently developed a novel experimental paradigm that has the potential to disentangle the two levels described above. This approach has been successfully employed to study gaze- and action-perception as well as closely related sensory-motor processes such as automatic gaze-following (Teufel et al., 2009, 2010a,b; Moore et al., in press). To illustrate this methodology, let us consider a study that applied this approach to study gaze-adaptation. Following extended exposure to a stimulus, many neural mechanisms coding the properties of that stimulus become less responsive. This adaptation biases the responses of populations of neurons recruited to perceive subsequently presented stimuli, and thereby leads to specific and measurable distortions of perception (Frisby, 1979; Webster, 2011). These “aftereffects” of adaptation can thus be used to probe the representational and functional structure underlying perception. Importantly, they are not only seen in relation to low-level visual properties such as orientation and direction of movement but also in high-level social perception of faces and facial gestures (Thompson and Burr, 2009; Webster and Macleod, 2011).

In the gaze-adaptation paradigm developed by Calder and colleagues (Jenkins et al., 2006; Calder et al., 2008), observers are exposed to pictures of faces gazing in a specific direction. Subsequent to this adaptation, the observers' perception of other people's gaze direction is biased toward the opposite side. In other words, adaptation to leftward gazing faces leads to a subsequent bias in gaze-perception to the right and adaptation to rightward gaze leads to a subsequent leftward bias. These gaze-direction aftereffects indicate the existence of distinct populations of neurons in the human brain that are specifically tuned to different gaze-directions. Moreover, a recent study combining adaptation and fMRI localized these neurons in the human STS (Calder et al., 2007), lending support to the notion that the processes demonstrated with the gaze-adaptation paradigm are akin to those shown in single-unit recordings employed in macaques (Perrett et al., 1992).

In order to disentangle a purported role of ToM in gaze-perception from contributions of lower-level perceptual processes, we recently modified this paradigm in various ways (Teufel et al., 2009). First, the static pictures of previous studies were exchanged for short video clips of two people; one was used as the adaptor (i.e., the stimulus to which the observer adapted) who was looking either to the right or left, and the second person was used as the test stimulus. An elaborate deception procedure convinced observers that these clips showed real people on-line via a live camera-link to an adjoining room similar to video conferencing. Moreover, the specific mental states that observers attributed to the person used as the adaptor stimulus were directly manipulated by the use of two pairs of goggles. The lenses of these goggles were highly mirrored and therefore appeared identical from the perspective of an onlooker. From the perspective of the person wearing them, however, one was transparent so that the person could see and the other was opaque, thus blindfolding the wearer (Novey, 1975; Heyes, 1998). Prior to the experiment, observers experienced these visual properties for themselves, so that when they saw another person wearing them, one pair signaled that the other could see and the second pair signaled that the other could not see. Observers were adapted to short video clips of a person looking in a specific direction, believing that this pre-recorded video showed—via a live camera-link to the next room—a real person who was either able or unable to see. Gaze-direction aftereffects were subsequently measured by the observer's bias in perceiving another person's eye-orientation. Note that across observers, bottom–up sensory input was identical between conditions in this design; the only aspect that changed was the observer's belief of the adaptor's ability to see. The results of this study effectively reduce to two basic findings. First, significant aftereffects were found in both conditions. Second, aftereffects were significantly larger when observers believed that the person they adapted to was able to see than when they believed this person was unable to see through their (opaque) goggles.

According to our analysis in the previous sections, the stimuli used in this experiment resemble social stimuli as encountered in a real social interaction in two different ways: first, the stimulus configuration of a face looking to the left or right resembles perceptual properties as encountered in everyday social settings and should trigger information processing of mechanisms tuned to these properties. Second, independent of perceptual properties, the stimulus has a deeper social dimension in that the observer has beliefs regarding the other's ability to see or not see. We hypothesize that the two different aspects of the results map onto these different stimulus levels and the respective types of information processing. Recall that adaptation effects were found in both conditions. We argue that the first finding is a result of processes in the visual system that are specifically tuned to the perceptual properties of another person's gaze direction (Jenkins and Langton, 2003; Calder et al., 2007, 2008). In addition to these processes, which are most likely shared with nonhuman primates and possibly other social mammals, humans possess an information processing system that prioritizes perceptual information on the basis of its social relevance. This is reflected in the modulatory effects that mental-state attribution exerted on gaze-processing. Note that only the use of an innovative procedure such as the one we employed allowed us to demonstrate the difference between these two types of information processing.

In order to further support our hypothesis regarding the importance of appropriate stimulus qualities to tease apart the various levels of information processing in social perception, we conducted an experiment with a procedure identical to the previous study except that we informed observers about the fact that they watched videos rather than convincing them that they watched a real person. They were asked to keep in mind that at the time of filming the videos, the person they adapted to was either able or unable to see dependent on the glasses they were wearing. Based on the assumption that social information processing at the level of stimulus properties is indifferent to the high-level conceptualization of the perceptual situation the observer is faced with, we predicted that gaze-direction aftereffects should be seen in both conditions. Furthermore, if information processing at the level of mental-state attribution is sensitive to whether the observer believes they are watching a video or a real person, by contrast to our previous study, we expected the adaptation effects not to be modulated by the condition. Comparing the current findings to a re-analysis of our previous findings, we should find a significant interaction between the two experiments and the two conditions.

## Materials and methods

The materials and methods used in this study were identical to those in Experiment 2 of a previous study conducted by the authors (Teufel et al., 2009) except for two aspects. First, in the current study, no deception procedure was employed; observers thus knew they watched videos rather than believing they interacted with a real person. Second, two novel experimenters ran the experiments.

### Observers

Fifty-six healthy observers with normal or corrected-to-normal vision completed the experiment. Observers were between 18 and 25 years old; the sample was roughly balanced with respect to gender. Given that we did not intend to analyse effects of age and gender, more specific information was not retained. Observers gave written consent, received payment and were fully debriefed after completion of the experiment. The study was approved by the local ethics committee.

### Apparatus and materials

As described in more detail in a previous paper (Teufel et al., 2009), we used two different pairs of goggles. One pair, either with a blue or yellow frame, was transparent from the perspective of the person wearing the goggles so that they could see through the goggles; the second pair, with a blue or yellow frame color to distinguish it from the first pair, was completely opaque, thus blindfolding the wearer. Both pairs had highly mirrored lenses that appeared identical from the perspective of an onlooker, similar to highly mirrored sunglasses. Observers received first-person experience of the translucent or light-blocking properties of these goggles prior to the experiment. When they saw another person wearing the goggles, one pair therefore, signaled that the other could see and the other pair indicated that the other person was not able to see.

### Stimuli

The stimuli used in this experiment are described in more detail in a previous paper (Teufel et al., 2009). All video images subtended 20° horizontally by 16.2° vertically of visual angle and were presented in the center of a Sony Trinitron Multiscan E530 screen using an Apple Mac Mini Computer running PsyScope-X software. By contrast to our previous study, interstimulus and intertrial intervals just showed a blank screen with a fixation cross.

**Figure 1:**
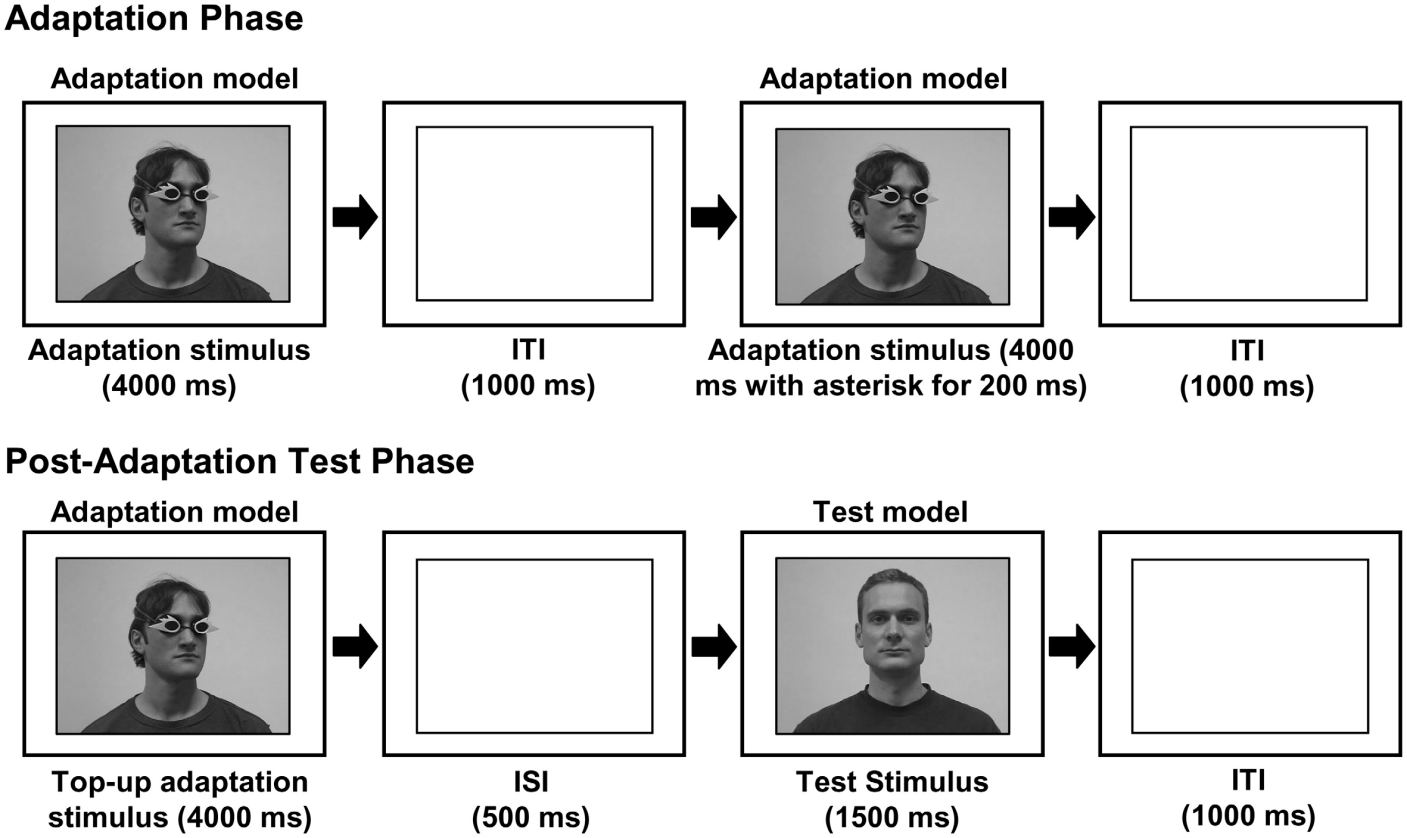
**A schematic illustration of the experimental procedure (ITI—intertrial interval; ISI—interstimulus interval).** The experiment consisted of an adaptation phase and a post-adaptation test. During the adaptation phase, which consisted of repeated presentations of an adaptation stimulus, observers were adapted to a specific gaze-direction (as indicated by head-orientation). This adaptation block was then followed by a post-adaptation test, in which observers' gaze-perception was measured. Each post-adaptation test trial consisted of a test stimulus gazing to the left, straight ahead, or to the right, preceded by a top–up adaptation stimulus.

The video-sequences used as adaptation and top–up adaptation stimuli showed a head and shoulders view of a male adaptation model, wearing mirrored goggles and with the head rotated 25° to the left or right with the shoulders in frontal view (Figure 1). Each adaptation and top–up adaptation stimulus consisted of a new video sequence. Most clips were highly similar but on a few the adaptation model moved in a specific way (cheek scratching, coughing etc.).

The test stimuli consisted of a head and shoulders view of a male model with the head pointing straight ahead and the eyes averted 5° to the left, straight or 5° to the right (Figure 1). We used 10 different videos of each of the different gaze-directions and the same 30 stimuli were used in all sessions. All test stimuli were highly similar.

### Procedure

Each observer participated in two conditions: A “Seeing” condition, in which the adaptation model wore the goggles that the observer had previously experienced as transparent and a “Non-Seeing” condition, in which the adaptor wore the opaque goggles. The order of conditions and the color of the goggles were counterbalanced across observers. Within each condition, we used a paradigm with an adaptation phase prior to a post-adaptation acuity phase. Each adaptation phase consisted of 24 trials in which the adaptation videos were shown in random order. Each clip was presented for 4000 ms with a blank screen for 1000 ms between clips (Figure 1). The observers were instructed to attend to the person on the screen and to keep in mind that, at the time of shooting the videos, the person was or was not able to see depending on the goggles they were wearing. As in our previous study, no further clarifications as to what the person in the videos was looking at were given. On half of the adaptation trials an asterisk appeared 400 ms after the start of the video in one of 12 different locations distributed over the eye region of the adaptor's face for 200 ms. Observers were instructed to press the space-bar as quickly as possible in response to the appearance of this asterisk. Response time measurements allowed us to estimate the amount of attention allocated to the face shown in the videos in the Seeing compared to the Non-Seeing condition. In total, each observer participated in four adaptation phases: one adaptation phase in which the adaptation model on the screen was turned to the left and one in which they were turned to the right in both the Seeing and the Non-Seeing condition.

A post-adaptation acuity test directly followed each adaptation phase. Each trial comprised of a top–up adaptation video in which the adaptation model was shown turned in the same direction and wearing the same goggles as during the previous adaptation phase, followed by an interstimulus interval (ISI; 500 ms) and by a test stimulus (showing the test model facing straight on and gazing either 5° to the left, straight ahead or 5° to the right; Figure 1). Chosen randomly, 10 trials were left trials, 10 were straight trials and the remaining 10 were right trials. The observers were instructed to indicate the gaze-direction, left, direct, or right by pressing the respective buttons 1, 2, or 3 on the keyboard.

A training phase prior to the first adaptation phase in both conditions was used to ensure that participants were able to perceive the different gaze-directions shown in the test stimuli. This phase was identical to the post-adaptation acuity without the presentation of any top–up adaptation stimuli. Similar to our previous study, we excluded three observers whose performance was more than two standard deviations below the group in the training session (less than 54% correct), assuming that this low performance indicated general problems in judging gaze-direction. Average acuity rates and standard deviation of the remaining 53 observers in the two training sessions were 83% (range ± 12%). Further details of the experimental procedure can be found in (Teufel et al., 2009).

### Analysis

Following Teufel et al. (2009), we calculated an overall adaptation score by assigning a 1 to left-responses, 0 to straight-responses and −1 to right-responses. These scores were summed within the test phase of each condition (separately for Seeing and Non-Seeing conditions and for adaptation to leftward gaze vs. rightward gaze), yielding a measure of mean gaze-direction judgment. The greater the tendency to perceive gaze-direction as rightward, the more negative this overall bias score was, and the more gaze was perceived as leftward, the more positive it was. These composite scores were analysed using a 2 × 2 repeated-measures ANOVA with factors of Condition (Seeing or Non-Seeing) and Adaptation Side (Left and Right). A note of caution in interpreting these results was necessary in that the residuals violated normality assumptions (Shapiro-Wilks Tests *p* < 0.05). Although we were confident that our large effective sample size meant that the analysis should be robust to them, we conducted a non-parametric equivalent to this analysis by directly calculating aftereffects separately for Seeing and Non-Seeing Conditions—achieved by subtracting the composite score for the Right from the Left adaptation condition in each case. These aftereffects were then compared for Seeing vs. Non-Seeing conditions using a Wilcoxon test.

To set this result in context, we planned to re-analyse data from our previous study (Teufel et al., 2009) and directly compare those aftereffects to the ones calculated for the current study. We were specifically interested in comparing the modulatory effect of mental-state attribution on gaze-direction aftereffects when participants believed they were viewing a “live” person with current mental states (our previous study) vs. when they knew they were watching videos (the current study). Given that there was a considerable difference in sample size between the two studies (16 vs. 56) that would exacerbate the effects of a marginally significant violation of homogeneity of variance (Levene's test: *p* < 0.05 for both Seeing and Non-Seeing levels of the factor Condition) and the non-normality of the resulting residuals (Shapiro Wilks tests *p* < 0.05), it was obvious that a mixed-ANOVA design might not be reliable. Accordingly, for that analysis, we subtracted the aftereffects for the Non-Seeing condition from those of the Seeing condition to serve as an index of the influence of Condition (Seeing vs. Non-Seeing) on the effects of adaptation: positive scores would indicate larger effects of adaptation in the Seeing than the Non-Seeing condition as had previously been found (Teufel et al., 2009). We performed this calculation on the scores for the current study and the previous study and then compared them using a Mann-Whitney *U*-test.

## Results

**Figure 2:**
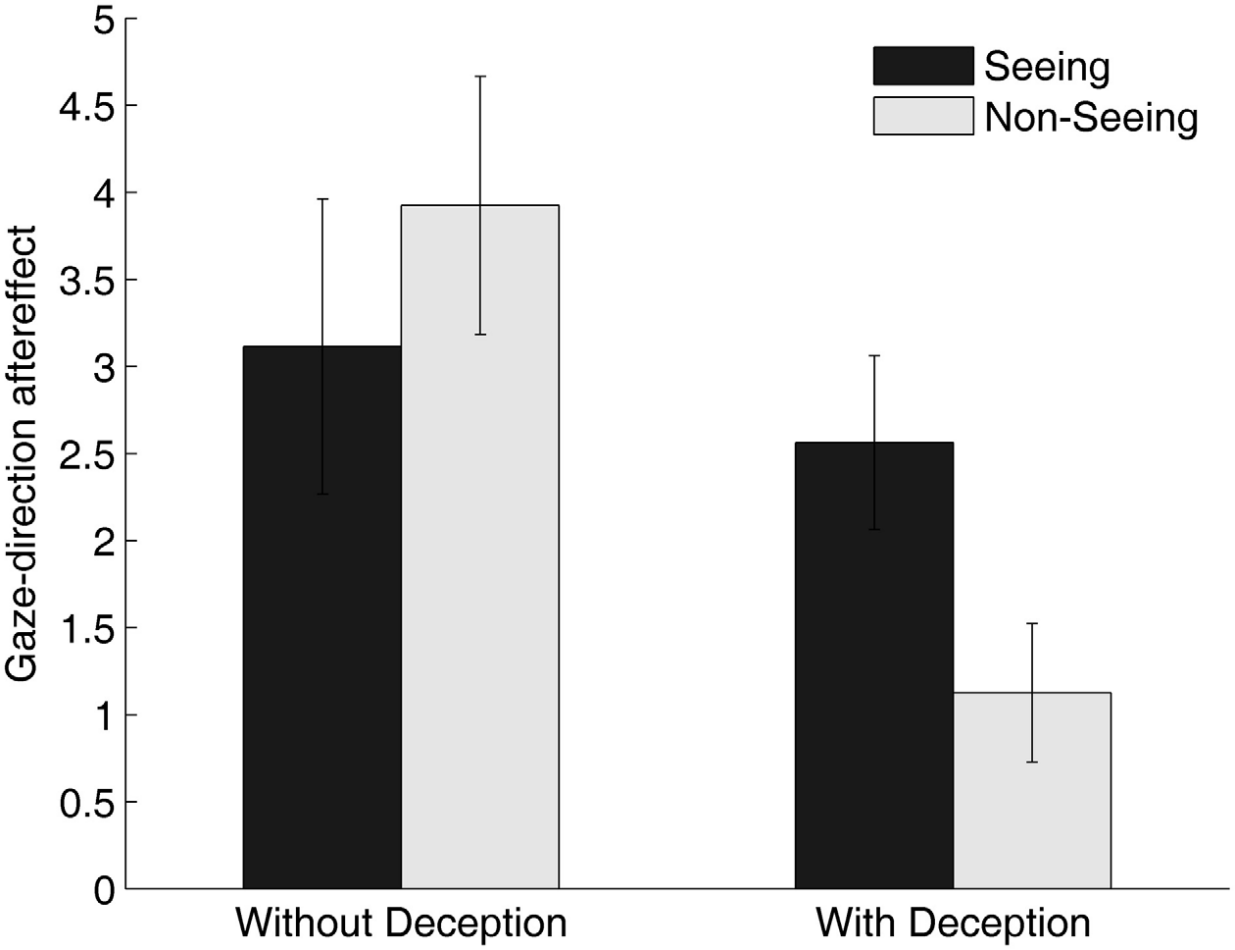
**Gaze-direction aftereffects in the Seeing and Non-Seeing condition.** The left panel shows the results of the current study (without deception); in the panel on the right, results from the second experiment of our previous study (with deception; Teufel et al., 2009) are re-plotted for comparison purposes. Aftereffects were calculated by subtracting the judgment scores for the rightward adaptation from the leftward adaptation conditions. Larger gaze-direction aftereffects indicate a larger difference in the influence of adaptation on subsequent gaze-direction perception between the rightward vs. the leftward adaptation conditions.

Mean gaze-direction aftereffects are plotted in Figure 2, separately for the current study (left panel) and our previous study (right panel; Teufel et al., 2009). As is clear from viewing the figure, there were robust gaze-direction aftereffects in the current experiment that seemed not to be modulated by the condition. A repeated-measures ANOVA [Condition (Seeing vs. Non-Seeing) × Adaptation-Side (Left vs. Right)] confirmed this impression: there was a significant main effect of Adaptation-Side [*F*_(1, 52)_ = 50.19, *p* < 0.001] but no main effect of Condition [*F*_(1, 52)_ = 1.45, n.s.] and no Condition × Adaptation-Side interaction [*F*_(1, 52)_ = 1.69, n.s.]. However, as described above, the residuals from that analysis were not normally distributed and transformations did not resolve this problem. Accordingly, we subtracted scores in leftward adaptation from rightward adaptation scores in each condition to provide an index of aftereffect magnitude and compared these in the Seeing vs. Non-Seeing conditions using a Wilcoxon test; this yielded a virtually identical result to the ANOVA, bolstering our conclusions from that first analysis (Z = –1.3, *p* = 0.19). The RT data did not differ significantly in the two conditions (380 ms vs. 369 ms for Seeing and Non-Seeing conditions, respectively, *t*_(52)_ = 1.793, *p* > 0.05).

When we compared the magnitude of the adaptation effects in each condition to the data from Teufel et al. (2009) a different pattern of results emerges in the two studies. Statistical analysis of these data indicated that the condition (Seeing vs. Non-Seeing) had a significantly different influence on gaze-adaptation in the two studies (*U* = 280, *p* < 0.05). Indeed, the evidence from the current sample was that, by contrast to our previous experiments, condition had no effect on gaze-adaptation. The differences in RT between the Seeing and the Non-Seeing conditions was not significantly different in the two experiments (*U* = 363, n.s.).

## Discussion

In the current article, we put forward a framework that addresses the extent to which stimuli employed in conventional social perception experiments address the crucial dimensions observers encounter in social interactions in the real world. We argue that, at the most basic level, it is important to distinguish between (1) the spatiotemporal properties that set apart certain social stimuli such as faces or biological motion from other object classes, and (2) the deeper social dimension that is characteristic of stimuli in real-life social interaction because they indicate other people's mental states. Moreover, we support this account with empirical data from an experiment in which we employed a modified version of the gaze-adaptation paradigm (Teufel et al., 2009). In combination with our previous findings, the results of this experiment indicate that whereas conventional stimuli can be used to study the functional organization of those parts of the visual system that are particularly tuned to socially-relevant spatiotemporal stimulus characteristics, they are not well-suited to address processes in social perception that are sensitive to higher-level conceptualizations of those stimuli in terms of the underlying mental states. We demonstrate aftereffects in gaze perception after extended exposure to a specific gaze direction, indicating adaptation of specific populations of neurons tuned to the adaptor's direction of gaze. Yet, despite using identical stimuli as a previous study (Teufel et al., 2009) we found no indication of a modulatory effect on gaze-direction aftereffects by attributed mental states. This is most likely due to the fact that observers knew they were watching videos of other people rather than believing that they interacted with a real person as in our previous study. In the remainder of this article, we discuss these findings as well as potential implications of our framework for social perception research more generally.

On the one hand, the finding that no modulatory effects of mental-state attribution were evident when observers knew they were watching videos ties in nicely with previous findings. To our knowledge, there are no other studies that directly scrutinize the role of (believed) presence or absence of another person for ToM processes in social perception similar to the current study. However, a couple of other researchers have demonstrated the importance of the believed or actual presence of another person in perceptual processing of faces (Pönkänen et al., 2011) or another person's actions (Wohlschläger et al., 2003a,b). These studies indicate that different information processing mechanisms are recruited when observers believe the stimulus they see is a real person that is present at the time of viewing compared to when they believe that they are watching a representation of an absent other. The findings of the current study, in combination with our previous results (Teufel et al., 2009), might provide an explanation of why online presence has an effect on social perception. In particular, we hypothesize that actual or believed online presence of another person heightens the tendency of observers to attribute mental states to the viewed stimuli. Mental-state attribution might in turn have top–down effects to prioritise the perceptual processing of specific socially-relevant information (Teufel et al., 2010b).

Whereas this proposal might gain some plausibility from the current literature, it is important to note that there are studies that have demonstrated differential effects of ToM-like processes on perception and associated sensory-motor responses when static pictures or animations were employed (e.g., Stanley et al., 2007; Liepelt et al., 2008; Nuku and Bekkering, 2008; Longo and Bertenthal, 2009; Wiese et al., 2012). We use the term “ToM-like” in this context because, as we have argued in the introduction, observers know that pictures, videos, and animations do not have mental states and these stimuli are, at best, ambiguous targets for mental-state attribution. For instance, in a gaze-cueing paradigm, Wiese et al. (2012) presented pictures of a human or a robot to observers and, by means of instruction, manipulated the extent to which observers believed the pictures represented a human gazing to the left or right, a humanoid-looking puppet, a robot whose gaze-behavior was controlled by a human, or a computer-controlled robot. Gaze-following, the tendency of observers to attend to locations looked at by others, was found to be larger in the human and human-controlled robot conditions than the puppet and the computer-controlled robot conditions, consistent with the idea that observers' high-level conceptualization of the stimuli as intentional agents determined gaze-following behavior independent of visual properties.

This finding is in stark contrast to the results reported in the current study. Whereas we did not find a modulatory influence on gaze perception by ToM in response to videos of real people, Wiese et al. (2012) found such effects in response to pictures in gaze-following, a behavioral response closely associated with gaze perception (Teufel et al., 2010b). A possible explanation for this puzzling pattern of findings is that there is a gradient of the extent to which ToM processes are recruited by different types of stimuli. In other words, observers might attribute similar mental states to pictures of people as to live people, yet these attributions might be inherently volatile and inconsistent across individuals. From this perspective, the use of interactions with live people as stimuli or of elaborate deception procedures to create this impression simply serves to increase the tendency of observers to attribute mental states and to exert some control over this process.

This possibility highlights one of the caveats of the comparison between the current study and our previous study. In both studies, the Seeing and the Non-Seeing conditions differed only in the observers' belief about the stimuli and instructions given prior to the experiment thus provide the main tool of manipulation. The experimenter's ability to communicate adequately and to “convince” observers to adopt the desired conceptualization of the task is therefore a key variable in determining the results. The comparison of the current results with those of our previous study therefore has to be viewed in light of the fact that these studies were conducted by different experimenters. Whereas this is a potential confound of the comparison, it is reassuring in this context that the two experiments reported in our previous study (Teufel et al., 2009) were conducted by different experimenters and resulted in similar findings, and that in the current study, two experimenters each tested half of the observers and found identical results. In any case, given the importance of the instructions and the experimenter in this kind of paradigm, it will be necessary in the future to find a way of objectively quantifying how successfully the instructions lead the observer to adopt the desired conceptualization of the stimuli.

There is an alternative, non-mutually exclusive way to reconcile studies that suggest that the online presence of another person has an important influence on perceptual processing with those studies that suggest that ToM-like processes can be triggered even by pictorial representations. The ability to engage in ToM requires a diverse collection of different socio-cognitive processes (Frith and Frith, 1999, 2003; Adolphs, 2009; Apperly and Butterfill, 2009) and it is thus possible that viewing a real person or a picture of a real person might tap into different types or aspects of mental-state attribution. A prime candidate for what might render the online presence of another person so important is the potential for direct engagement and interaction. Whereas an observer might attribute mental states to a movie character or even to a static picture of another person, such stimuli do not provide the potential for interaction (see also Laidlaw et al., 2011). This account is in line with a more recent development in social cognitive neuroscience that emphasizes interaction as the crucial and much neglected variable in social information processing (Schilbach et al., 2006; De Jaegher et al., 2010; Bohl and van den Bos, 2012). It will be important to assess the extent to which mental-state attribution has differential effects on visual processing depending on whether the observer directly engages or has the potential to engage with the stimulus in comparison to when they adopt a detached third-person perspective of the other. At present, we know little about the importance of this factor, but a fully-developed framework of the different levels of social stimuli will have to incorporate this variable in the future; this research topic would surely benefit from a more detailed taxonomy of the different aspects of mental state attribution in question.

Methodologically, this point emphasizes the inherent tension between ecological validity and experimental control in social perception research. Most current research tools of social perception are borrowed from other areas of vision research and psychophysics. For good reasons, many of these designs have withstood the test of time and should be part of the toolkit of every researcher in social perception. However, we argue that social perception is special in that certain stimulus dimensions cannot easily be incorporated in standard paradigms. In other words, while standard designs using simple pictures as stimuli might provide accurate internal validity, the extent to which they approximate what the visual system is faced with in a real social interaction might be very limited. In fact, a similar point has recently been brought forward by Kingstone and colleagues regarding the ecological (in) validity of much of vision research more generally (Kingstone, 2009). We believe that the current challenge in social perception is to develop new paradigms with high ecological validity that do not sacrifice full experimental control.

The previous point is of particular interest when considering implications for clinical studies. Autism Spectrum Conditions (ASC) are a group of neurodevelopmental conditions where difficulties in social interaction and communication are defining characteristics. Despite being well-studied, the literature on social deficits in ASC shows many inconsistencies (e.g., Nation and Penny, 2008; Falck-Ytter and Von Hofsten, 2011). For instance, although individuals with ASC display difficulties in using information from another person's eye region to guide their social behavior and show a striking tendency to avoid the eyes in social interactions, this atypical gaze behavior is only inconsistently observed in a laboratory setting when viewing images or videos of faces or social scenes (Falck-Ytter and Von Hofsten, 2011). In fact, individuals with ASC tend to show normal performance on at least some typical social perception tasks involving eye gaze such as discrimination of gaze-direction (Baron-Cohen, 1995) and reflexive orienting to gaze cues (at least in some studies; see Nation and Penny, 2008 for review).

A hypothesis that could contribute to an explanation of the apparent inconsistencies between laboratory findings and real-world observations might be derived from our proposed framework. The visual system of autistic individuals might show, at least in part, normal mechanisms tuned to the specific spatiotemporal characteristics of socially-relevant stimuli. It is this property that conventional social perception paradigms with pictures, animations, or videos tap into and, given the assumption, it therefore is no surprise that no differences between patients and controls are found. Yet, due to a dysfunction in implicit top–down modulation of perceptual processing by mental-state attribution, autistic individuals might process these signals in a fundamentally different manner with respect to the deeper social dimension, and this might account for many of the difficulties seen in everyday interactions. A fundamental challenge therefore remains the development of new paradigms which more closely resemble real social situations in order to begin to address these issues. In the field of autism research, the case to do so is particularly compelling but progress has been slow in adopting more ecologically valid stimuli. Recent developments offer a tantalizing glimpse of the types of questions that may be asked of clinical disorders such as autism in the future (Redcay et al., in press).

In conclusion, we propose a framework in social perception research that highlights different ways in which experimental stimuli can match the crucial dimensions of social stimuli as encountered in real social interactions. In particular, we distinguish between the spatiotemporal characteristics of a stimulus and the deeper social dimension the stimulus has in terms of its potential to indicate another person's mental states. This framework has important implications for experimental design and clinically-relevant work. To date, the number of studies speaking to this issue is limited and provides a fascinating but puzzling mosaic.

### Conflict of interest statement

The authors declare that the research was conducted in the absence of any commercial or financial relationships that could be construed as a potential conflict of interest.
